# Z factor: a new index for measuring academic research output

**DOI:** 10.1186/1744-8069-4-53

**Published:** 2008-11-09

**Authors:** Min Zhuo

**Affiliations:** 1 Department of Physiology, Faculty of Medicine, University of Toronto, Medical Sciences Building, 1 King's College Circle, Toronto, Ontario, M5S 1A8, Canada

## Abstract

With rapid progress in scientific research activities and growing competition for funding resources, it becomes critical to effectively evaluate an individual researcher's annual academic performance, or their cumulative performance within the last 3–5 years. It is particularly important for young independent investigators, and is also useful for funding agencies when determining the productivity and quality of grant awardees. As the funding becomes increasingly limited, having an unbiased method of measuring recent performance of an individual scientist is clearly needed. Here I propose the Z factor, a new and useful way to measure recent academic performance.

## 

Of late, there have been continuous and intensive debates regarding the roles of the impact factor [1-7]. There are many reasons the misuse of the impact factor, sometimes called the 'impact factor fever', generates concern. First, the impact factor does not provide any evaluation for the quality of science in the research articles. Although the majority of papers published in high impact journals are generally novel and of highest quality, there are a few 'bad apples'. Second, the impact factor represents the mean citation rate of papers published in one journal, and it does not represent the citation of the specific article itself. A paper published in a low impact factor journal may end up well cited, while conversely, a paper published in a high impact factor journal may garner very few citations. Despite these limitations, it is still generally agreed upon that it is much harder to publish an article in a journal with an impact factor of greater than 10 than a journal with an impact factor of 1. It is therefore unfair to claim that the impact factor is useless or only doing harm to the academic community.

Considering the increase in scientific activity in recent years, it is important to have objective criteria for evaluating academic performances when considering, for example, new faculty recruitment, annual performance and tenure evaluation, and funding decisions. Unlike commercial businesses, the broad spectrum of academic research activities render effective evaluation by administrators or funding agencies to be rather difficult, especially those who are involved in 'basic' research. In most cases, published papers are the only considerations for evaluation. It is important to have a consistent standard to evaluate these papers, as most will not be translated into commercial activities or be awarded with the Noble prize for a few decades to come, if at all. However, it is these 'basic' researches that lay the foundations and holds hope for future drug discoveries and treatments of human diseases.

What is the best way to evaluate scientific performance? I still recall one department head claiming that good science in his department was defined by whether he thinks the researcher is doing good science, not by where the faculty member has published papers (including in high impact factor journals), nor by how many papers he or she has published. Although there may be some merit in holding to such a high standard, this would require the department chair, university dean or government funding agency to carefully read and evaluate the individual papers. Considering the increasing number of disciplines in scientific research, this has become almost impossible. And what about the panel of experts? Can we always trust the experts' evaluation? Yes and no. First, it depends on who is sitting on the review board. For example, while the NIH study sections have been seen as the best review system for evaluating scientific proposals, they have also been consistently under criticism for many years. Second, it is impractical for each department to invite experts to review its faculty members annually. It must also be pointed out that it is impossible to remove the human aspect with the use of experts. What happens if a faculty member is a long-term collaborator or a family friend of an expert reviewer? What happens if a faculty member proposes a model that goes against a reviewer's long-held theory? In reality, our decisions are affected by culture, religion, race, friendships and personal interests. Thus, I believe that there is a need for a better measurement that should be used to evaluate a researcher's performance. Here I propose a new index to evaluate the active performance of an individual scientist, and I will explore how such an index can be used in combination with current impact factors and H factors to attain a better assessment of one researcher's productivity.

There are currently three major factors that are used to evaluate academic performances: the number of publications, the impact factor of those journals, and the H factor (see Table 1). For the number of publications, it is the number of peer-reviewed research articles that are ordinarily counted. Review articles and meeting abstracts are often put in a separate category since most of them are invited or not reviewed. The journal impact factor is the average number of times the articles from the journal published in the past two years have been cited. The journals with highest impact factors are ***Cell*, *Science ***and ***Nature***, ranging between 30 and 40. Publishing in these journals has often been considered to be the best academic achievement and news worthy. Lastly, the recently introduced [8] and not commonly used method is the H factor. It overcomes the differences in subfields of research areas by quantifying both the actual scientific productivity (the number of publications) and the apparent scientific impact of a researcher. The index is based on the number of papers the scientist has published and the number of citations that they have received in other people's publications. For example, to get an H factor of 20, one must have published at least 20 papers, which must be cited 20 times.

**Table 1:** List of factors used for the measurement of researchers' performance

**Parameters**	**Definition**	**Disadvantage**
***N***	The total number of publications	Does not consider the quality of the study; co-authorship

***I***	The impact factor of the journal where the paper is published	Does not consider the productivity; co-authorship

***H***		Too early for young investigators

***Z***	The new measure integrates both the impact factor of the journal and the productivity	---

## Problems to be tackled

Due to space limitations, journals such as ***Science ***and ***Nature ***mostly select papers on 'hot' topics of science. Quite often, what constitutes a hot topic changes over the time. It can also be influenced by the scientific policy of the current government, as often seen in ***Science***. Due to the space limitation (the way to get such high impact factors, as suggested by some experts) [see [2]] and the focus on hot topics, scientists commonly complain that some papers are not published in high impact journals simply because the paper's topic is not 'hot' enough. Additionally, many papers in highly profiled journals face intense scrutiny, and indeed, a few are un-reproducible or are downright fabricated. Papers in low impact journals usually do not receive such careful examinations. High impact journals also typically require novelty, and it is difficult to switch gears enough to publish in those journals, particularly if your focus has been on your own area of research for a long period of time. While there is nothing wrong with publishing in lower impact journals, the problem occurs when some scientists strive to publish in high impact journals by constantly changing their research topics. Many outstanding scientists, including some who have spent decades becoming established in their field, feel concerned or even depressed because they lack high impact papers in recent years. To help overcome this 'mental' burden, I suggest the use of a new index factor, called the Z factor. The Z factor considers both the number of publications and the impact factors of the journals in which they were published.

There are three factors that are used to generate the Z score: P, I and N. P is the contribution factor. In cases where the individual is the first/equal author or the corresponding last author, P = 1. In collaborative efforts, P = 0.3 (where one is neither the first nor the last author). P = 1 if the paper specifically notes that there are two equal corresponding authors. Therefore, it is important for collaborative principle investigators (PIs) to indicate such equal contributions in a paper. Next is I, the impact factor of the journals where they have published within the last year. Since it usually takes 1–3 years for a single paper to be cited, it is impossible to obtain appropriate citations of papers which have only recently been published. Therefore, in order to perform annual evaluations, instead of using the actual and often unavailable citation of the individual papers, the impact factor of the journal where the authors have published in the last year is to be used. For example, for ***Nature***, I = 28.8 in 2007, and for ***Neuron***, I = 13.4. Lastly, N is the total number of papers published within one year.

$$\text{Thus},\text{ Z}=\sum_{\text{I}=1}^{\text{N}}\text{IiPi}$$

Table 2 contains examples of evaluations of 4 professors from Harvard University (2 full professors and 2 junior assistant professors). For Professor A, in 2007 (with H factor at 24), only one paper has been published in a journal with I = 13.4, and therefore the Z score is 13.4. For Professor B, in 2007 (H = 70), six papers have been published in various journals (I ranges from 2 to 8), and thus the Z score is 49.0. C is a junior assistant professor (with H factor at 16); with three papers published, the Z score is 57.8. Therefore, the Z score will allow comparisons between senior and junior faculty members. This is potentially quite useful, since most funding agencies such as NIH and CIHR do not treat them separately.

**Table 2:** Performance examples of four professors from Harvard University during the last 12 months (based on data from Pubmed and Web of Science)

	**H**	**N**	**Z**
***Professor A***	24	1	13.4

***Professor B***	70	6	49.0

***Assistant Professor C***	16	3	57.8

***Assistant Professor D***	17	0	0

In addition to counting the number of publications, the Z factor gives significant credit to those published in high impact journals. And unlike the H factor, which requires a waiting period of at least 2–3 years after publication to reflect any change, the Z factor can be calculated annually. In my opinion, this method will be very useful in evaluating one's academic achievements annually.

The Z factor can be used in other ways. For example, one can use the Z factor to evaluate a faculty member's achievement in their specific research field over a period of time (say 3–5 years). By using 2 or 3 keyword as defined by him or her as the major focused topics, one can generate a list of published papers. The Z factor based on these papers will give a nice measurement for his or her recent achievement in his research field. This will also help to eliminate possible erroneous accounting of researchers' publications, where their names appear on papers solely for lending the use of unique elements or methodologies to other investigators.

Also, the Z factor can aid in evaluating the research quality or originality of faculty members, notably if one faculty member always needs numerous papers (larger N) to catch up to the same Z score of another faculty member. In those cases, additional scientific review of published papers may be required. More importantly, the Z factor does not bias against those individuals who make basic and fundamental contributions to the established fields. Three papers published in journals such as ***Molecular Pain ***with impact factor of 4.13 can accrue to a similar Z score as one article published in a higher impact journal such as ***Nature Neuroscience ***(I = 14). Publishing three papers in Molecular Pain in three years is a reachable goal for many good pain researchers, but publishing one paper in Nature Neuroscience every three year can be an uncertain goal even for top neuroscientists. The Z factor will give individual scientists more freedom to focus on their own research interests, rather than having to change their research topic and chase after so-called 'hot topics' in their attempts to publish in high impact journals. More over, the use of Z factor will help to cool the fever for the impact factor, and encourage the publication of novel scientific findings [see [4]].

In summary, the Z score may serve better than the current methods of assessing achievement, which simply count the number of published papers, those published in high impact factor journals, or merely counting on the assessment from review members.

## Competing interests

The author declares that they have no competing interests.
